# Hepatitis B: Prevalence and occult infection in HIV-infected patients

**DOI:** 10.1590/0037-8682-0533-2018

**Published:** 2020-01-27

**Authors:** Samira Julien Calux, Vanessa Cristina Martins Silva, Adriana Parise Compri, Marcílio Figueiredo Lemos, Ana Paula de Torres Santos, Isabel Takano Oba, Maria Cássia J. Mendes-Correa, Regina Célia Moreira

**Affiliations:** 1 Laboratório de Hepatites Virais, Centro de Virologia, Instituto Adolfo Lutz, São Paulo, SP, Brasil.; 2 Divisão de Laboratório Central, Hospital das Clínicas, Universidade de São Paulo, São Paulo, SP, Brasil.; 3 Departamento de Doenças Infecciosas, Universidade de São Paulo, São Paulo, SP, Brasil.

**Keywords:** Prevalence, Occult hepatitis B infection, HBV, HIV

## Abstract

**INTRODUCTION:**

HBV and HIV have identical transmission routes. The aim of this study was to determine the prevalence of HBV in HIV patients and to detect the presence of occult HBV infection.

**METHODS:**

All samples were tested for serology markers and using qPCR.

**RESULTS:**

This study included 232 individuals, out of which 36.6% presented with HBV markers and 11.8% presented with HBsAg or HBV-DNA, including 3 patients that showed OBI.

**CONCLUSIONS:**

We observed a high prevalence of HBV among HIV patients. In addition, the results suggest that OBI can occur in patients with serological profiles that are indicative of past infection. Therefore, the application of molecular tests may enable the identification of infections that are not evident solely based on serology.

Hepatitis B virus (HBV) infection is a global public health issue. An estimated 240 million people are chronic carriers, and acute or chronic HBV infections result in approximately 887,000 deaths per year. In the Amazon region and southern regions of Eastern and Central Europe, chronic infections are prevalent among the adult populations. Due to vaccination programs in South America and Oceania, the prevalence rates of chronic HBV infection are less than 2%. In Western Europe and North America the burden of chronic infections is less than 1%^1^.The goal of OMS is to eliminate HBV through efficient vaccination programs by 2030.

Globally, approximately 35 million people are infected with HIV. The African continent, particularly sub-Saharan Africa, is the most affected region with an average 25.7 million people infected with HIV. Amongthe HIV carriers, at least 7.4% are co-infected with HBV^2^.

The high prevalence of co-infection is explained by the similarity in the transmission routes of hepatitis B virus and HIV^3^.The progression of chronic hepatitis to cirrhosis, hepatocellular carcinoma, and terminal stage liver disease is faster in individuals infected with HIV than in those infected with HBV alone. Therefore, co-infection can increase the morbidity and mortality compared with an HBV mono-infection^4^. 

The laboratory diagnosis of HBV infection depends on the serological detection of total anti-HBc, HBsAg, and other markers that may be used to monitor the infection and evaluate immune response^5^. In addition, molecular assays are used for diagnosis in children and monitoring of HBV infection.

In some cases of infection, low concentrations (lower than 200 IU/ml) of HBV DNA is detected in the serum or plasma of patients that tested negative for presence of HBsAg.This is a feature of occult Hepatitis B infection (OBI), which is described as the presence of HBV DNA in blood, at undetectable levels of HBsAg (with or without anti-HBc and anti-HBs), outside the pre-seroconversion window period^6^.This condition is reported frequently in HIV-infected patients, particularly among those that are treatment-naïve^5^. 

Owing to differences in sensitivity and specificity between different detection methods, the prevalence of OBI is variable worldwide among various categories of individuals^6^. In Northern countries where the prevalence of infection is below 5%, and the prevalence of chronic infection less than 1%, the prevalence of OBI does not exceed 5%^7^. In contrast, OBI is observed to affect 4-24% of the population in India, Taiwan, Japan, and Sardinia. In West Africa, approximately 5% of total HBV DNA carriers are HBsAg negative^8^.

The aim of this study was to determine the prevalence of HBV and detect presence of OBI, in a group of HIV patients. 

This study included HIV-infected patients, receiving follow up care at outpatient service that supports patients living with HIV/AIDS (SEAP) in São Paulo city. Between June 2013 and May 2014, two vials of peripheral blood were collected from each patient. Ethylenediaminetetraacetic acid (EDTA) was added to the samples. The blood samples were tested for the presence or absence of HBsAg, Anti-HBs, and total Anti-HBc antibodies. The tests were performed according to the manufacturer’s instructions (DiaSorin; Saluggia-Vercelii, Italy).

All the samples were subjected to HBV quantitative real time polymerase chain reaction (qPCR), according to the manufacturer's instructions (Abbott RealTime HBV, Des Plaines, IL, USA).To eliminate the pre-conversion window period, the PCR positive samples were tested for the presence of Anti-HBc IgM usinga Cobas® Anti-HBc IgM Cobas® -Roche Diagnostics kit (Mannhein, Germany). 

Blood samples were collected only after the written consent form was signed by the patient. This study was approved by the Ethical Committee in Research from Instituto Adolfo Lutz (CEPIAL#186.915).

This study enrolled 232 patients. HBV markers, wither in isolation or in association with other markers, were detected in 65.5% (152/232) of the patients.

Hepatitis B was detected in 36.6% (85/232) of samples with exposure markers. Out of these, evidence of chronic infection and previous contact with HBV was indicated in 8.2% (7/85) and 91.8% (78/85) of the patients, respectively (Table 1).

**TABLE 1:** Serological profiles identified in the study group.

Serological profile	Serological Markers			Total of samples (%)	
	HBsAg	Anti-HBs	Total Anti-HBc		
Total Anti-HBc only	Not reagent	Not reagent	Reagent	25	(10.8)
Anti-HBc and Anti-HBs	Not reagent	Reagent	Reagent	53	(22.8)
Anti-HBs only	Not reagent	Reagent	Not reagent	67	(28.9)
HBsAg and Total Anti-HBc	Reagent	Not reagent	Reagent	3	(1.3)
HBsAg only	Reagent	Not reagent	Not reagent	3	(1.3)
All tested markers	Reagent	Reagent	Reagent	1	(0.4)
No markers	Not reagent	Not reagent	Not reagent	80	(34.5)
Total				232	(100.0)

*Occult hepatitis B infection.

The viral DNA was detected in six samples, of which two were quantified . HBV DNA was detected in four samples. However, the concentration of HBV DNA was below the limit of quantification (10 IU/ml). The PCR positive samples tested negative for presence of anti-HBc IgM. The absence of serological markers of acute infection and the presence of HBV DNA indicated the presence of OBI in 3 patients (Table 2).

**TABLE 2:** Samples with detected HBV-DNA.

Sample ID	HBV-DNA (UI/mL)	Serological Group	Presence of OBI*
122	<10	HBsAg	No
112	462	HBsAg and Total Anti-HBc	No
105	<10	HBsAg and Total Anti-HBc	No
185	30	Total Anti-HBc isolated	Yes
167	<10	Total Anti-HBc isolated	Yes
187	<10	Total Anti-HBc an Anti-HBs	Yes

The values of prevalence and co-infection of HBV in HIV-infected patients observed in this study were similar to those reported in the previous studies conducted in Brazil. A study in São Paulo indicated that 38.6% of patients tested positive for total anti-HBc, and 5.7% of patients tested positive for HBsAg reagents^9^. In Ribeirão Preto, the overall prevalence of HBV infection was observed to be 40.9%, with 8.5% testing positive for HBsAg, 39.7% testing positive for total anti-HBc, and 8.5% co-infected^10^. In a study in the Amazon region, the prevalence was 40.2% for resolved HBV infection, and 6.4% of patients tested positive for HBsAg^11^. In the state of Ceará, 23% of patients were previously exposed to HBV and 3.7% were co-infected^12^. Recently, in Goiânia, the presence of HBV exposure markers were detected in 33.5% of the patients, while the HBsAg marker was detected in 3.8% of patients.

Total anti-HBc was isolated from blood samples of two of these patients, while total anti-HBc associated with anti-HBs was detected in one patient.

These data suggest that OBI can occur in patients with serology suggestive of past HBV infection or in those presenting serological evidence of viral exposure.

In a study conducted in Nigeria with 188 HIV-infected patients, OBI was detected in 21 patients, resulting in a prevalence of 11.2%^13^Thesedata corroborate the results of this study.

The impact of OBI in the prognosis of HIV-infected patients requires further investigation. However, evidence indicates that the presence of HBV DNA may be a risk factor in the progression of liver disease^5^ ^,^ ^8^. A research group in Italy monitored 86 HIV-infected patients for at least 6 months, out of which 17 patients presented with OBI. Moreover, acute exacerbation of hepatitis B occurred in 28 (32.5%) patients during the follow-up. This event was more frequently observed in 17 HBV DNA-positive patients than in the 69 HBV DNA-negative individuals. The authors suggest that OBI may be associated with a deteriorating liver disease in HIV patients^14^.

Results indicated a high prevalence of hepatitis B among HIV-infected patients. In addition, the results suggest that occult hepatitis B infection can occur in patients with serological profiles indicative of past infection. 
